# A human iPSC model of Tauopathies engineered for 4R Tau isoform expression endogenously develops late‐stage neuronal Tau pathology

**DOI:** 10.1002/alz70855_100354

**Published:** 2025-12-26

**Authors:** Angelika Dannert

**Affiliations:** ^1^ University Hospital, LMU Munich, Institute for Stroke and Dementia Research (ISD), Munich, Munich, Germany

## Abstract

**Background:**

Tauopathies, such as Alzheimer's disease and Frontotemporal Dementia, are common neurodegenerative diseases characterized by misfolding, hyperphosphorylation, and aggregation of Tau. Molecular mechanisms underlying Tauopathies are still poorly understood, in part due to a lack of human models endogenously developing major disease hallmarks. Adult Tau isoform expression contributes to Tau pathogenesis but is challenging to replicate in human stem‐cell‐derived systems, which impedes formation of late‐stage disease phenotypes and hence research on underlying mechanisms and drug development.

**Method:**

We developed a novel iPSC‐based cortical neuron model in which we altered Tau isoform expression to match adult human neurons. For this, we used a multi‐step CRISPR/Cas9 genome editing strategy that changes endogenous Tau isoform expression from the genomic MAPT locus.

**Result:**

We found that induction of adult human brain‐like 4R Tau isoform expression enables endogenous formation of late‐stage Tauopathy hallmarks in iPSC‐derived neurons engineered to contain synergistic Tau mutations. Neurons accumulated seeding‐competent, hyper‐phosphorylated, fibrillar Tau in tangle‐like structures. Furthermore, exclusive expression of mutant 4R in the absence of the 3R Tau isoform disproportionately intensified pathology, resulting in highly abundant Tau misfolding and aggregation. Finally, we found proof‐of‐principle that our model can be translationally applied both to test chemical disease modulators and evaluate a human Tau PET tracer.

**Conclusion:**

Collectively, our model enables novel investigations on endogenous mechanisms of human Tauopathy formation, suggesting a central role of 4R Tau isoform expression for pathogenesis in human neurons. Moreover, it may also serve as a platform supporting urgently needed development of disease‐modifying drugs.

